# An Assessment of the Role of Women Group in Women Political Participation, and Economic Development in Nigeria

**DOI:** 10.3389/fsoc.2019.00052

**Published:** 2019-07-12

**Authors:** Monica Adele Orisadare

**Affiliations:** Department of Economics, Obafemi Awolowo University, Ife, Nigeria

**Keywords:** women's group, women political participation, economic development, Nigeria, Osun state

## Abstract

The exclusion of women in politics has been identified in recent times as one of the major setbacks for economic development. Women's groups are a strong pillar for grassroots politics; and a drive for more women participating in politics at the grassroots still faces a lot of challenges, making it difficult for them to harness available opportunities for economic development. Thus, the opportunity therein for more women's participation in politics and women empowerment is yet to be exploited by the women's groups in Nigeria. This present study assesses the role of women's groups in politics, identifies their challenges and also explores its implication for economic development in Osun state, Nigeria. The study was carried out using primary data from forums and dialogues within women's groups, consisting of an average of thirty (30) members from ten (10) local government areas (LGAs) in Osun state, Nigeria. In addition, explorative methods using existing literature were also employed. Findings from the study indicate that women's groups do not have political agenda; mostly, their goals do not align with any political agenda, although their members accept appointments, and also enjoy government patronage. There was also an indication that there exists a high illiteracy rate among the members of the women's groups and most of them are not aware of existing National or International gender equality laws or affirmative action. Thus, it is difficult to participate in politics and contribute their voices to political issues. The study concludes that the present role played by women's groups at the grassroots level may not be adequate in encouraging more women's participation in politics and in influencing economic development. Thus, there is need to step up their activities and embrace political issues if they are to help more women participate and be relevant in politics. The study therefore suggests that more empowerment programs, especially in the area of decision making and participation in politics, should be targeted at women's groups at the grassroots levels by the governments and all stakeholders as a matter of priority.

## Introduction

The exclusion of women in politics has been identified in recent times as one of the major setbacks for economic development. The poor presentation of women in elective positions has been a major social development issue since the beginning of the current democratization process in Nigeria. Politics as a real-world phenomenon is gendered. The world over, core conditions of people's lives—including their health, education, security, as well as access to markets, public space, freedom of expression, and their work—are fundamentally shaped by their identification as being of a particular sex or gender group (Waylen et al., 2013). Evidence around the globe indicates the path for women to hold elected office was achieved not only through the efforts of individuals but with collective work through organizations. As identified by Mazur et al. (2016), participants in the women's movement, including individuals and groups, both informal and formal, are those who identify with women as a group, and are framed as women representing women whose ideas are expressed as overtly gendered. Beckwith (2000) viewed women's movements as a subset of sociopolitical movements focusing on women's gendered experiences. The author went on give women's sewing clubs as an example. Social clubs for women existed in the nineteenth century, but toward the end of the century an increasing number of women's groups began to form with political leanings. According to Mazur et al. (2016), it was not until the early 2000s that scholars started having a consensus on the meaning of women's movements for comparative purposes (Molyneux, 1985; Beckwith, 2000). Although, women's movements were treated by many early feminist scholars, such as Dahlerup (1986), Katzenstein and Mueller (1987), Ferree and Martin (1995), as a major analytical focus or variable; they tried to understand how changes in the nature of women's movements have influenced policy outcomes and in turn how these activities have affected the movements. In the earlier years, equal rights, may not have been the cause championed by women's movements but rather, in traditional African societies, they sought to protect a women's role of mothering and care-giving, a situation (Gouws, 2015) pointed out might not always be empowering. In recent times, however, the failure of the patriarchal dominated state to incorporated women's issues into governance has seen women's movement springing up around the globe and especially in Africa since the 1990s. Thus, women's movements have evolved overtime as a result of modernizing forces and processes of redefining the public (politics) and private sphere (household). There exists a long history of Women's movements in Western democracies, yet only a few conclusions can be drawn with confidence about their trajectories over the decades (Mazur et al., 2016). There are different variations of women's movements in existence in different regions of Africa, in terms of their timing, character, influence, and effectiveness.

Various organizations have helped educate voters, raise awareness about women's rights issues, lobby legislative bodies, and support women running for office. Even though some groups dissolved after their particular causes were no longer relevant, while other groups have been longer lasting, political groups for women nevertheless continue to play a role in politics today. Women's organizations, as observed by Gouws (2015), are spreading and networking across Africa on an unprecedented scale and creating gender-friendly laws and constitutions. Thus, in recent times, Women's movements which were hitherto dominated by organizations engaged in “developmental” activities including income-generation, welfare concerns, and home making skills, have evolved to become organizations lobbying for women in decision making position in politics: pressing for legislative and constitutional changes, and conducting civic education. In fact, women on their own went further to form political parties. From an account from Holm (1992), the reason was partly because women's concerns have not been adequately addressed by existing parties in the multiparty system. This is because in many instances women's political visions are different and do not align with existing parties structures and, thus, are not accommodated; in some cases, the women wanted to build more broad multiethnic and multi-religious constituencies than was possible with existing parties. Examples include: the National Party in Zambia in 1991 started by Dr. Inonge Mbikusita-Lewanika; the Zimbabwe Union of Democrats in 1999 by Margaret Dongo; and Kopanang Basotho in Lesotho formed by Limakatso Ntakatsane. Likewise, in Kenya, parties were headed by Charity Ngilu and Dr. Wangari Maathai; in Central African Republic, Ruth Rolland-Jeanne-Marie led a party; similarly in Angola, Amália de Vitoria Pereira led a party (Tripp, 1999). Women-led parties with broad male and female constituencies had sprang up in Zambia, Kenya and several other countries due to the reluctance of political parties to take steps to increase women's representation (Holm, 1992). In Nigeria however, although political parties are not yet led by women, women have been organizing themselves to redefine their position in the society since the pre-colonial period. In the last two decades women's organizations have witnessed a significant increase and a greatly diversified set of issues are being addressed. There are associations, cooperatives, trade groups, faith based organizations at local, state and national levels, and some with regional and international connections (Centre for Human Development, 2012). Despite the enormous achievements of the Nigerian women's movement so far, there remains a plethora of issues to be addressed urgently; a major one is the greater involvement of women in political and decision-making processes. The social and economic pressures which have become pronounced in the last two decades and the emergence of the male dominated democratic system are having a negative effect on the gains of the past. There is, therefore, a clear need to re-think the approach to women's empowerment and gender equality, especially in the area of increasing women's political participation.

This study lies on the premise that promoting women's participation through the role of women's groups in political dispensation is desirable based on equity, equality, and economic development. This premise is supported by Feminist empowerment approach which combines both Feminist theory and empowerment approach and Modernization theory. According to feminist theory, inferior status delegated to women is due to societal inequality, which is shaped by political, economic and social power relations, and women should have equal access to all forms of power (Turner and Maschi, 2014). Similar to the concept of empowerment, feminist analysis helps women to understand how they are oppressed and dominated and often inspires them to engage in efforts to bring about broader social change. Empowerment theories have appeared based upon feminist publications and theories. “Women's empowerment approach,” according to Razmi et al. (2018), is one of the most important theories proposed in recent years among both those theoretical and empirical. According to this approach, the women's share of parliamentary seats is one of the most important indicators for measuring Women's Empowerment (Razmi et al., 2018). It has been argued by scholars that an increase in the average level of human capital is preceded by an increase in inclusive incentives and policies for women in the labor force and enrolment in higher education (Stolt, 2013). Essentially, therefore, the ultimate goal of women's empowerment, which is a political process, is not just to change hierarchical gender relations but also to change all hierarchical relations in the society (Cook, 1997).

The modernization theory reckons that economic growth inevitably leads to social development and gender equality; authors dealing with conflict and with institutional design suggest that economic growth by itself does not follow an exclusive path. According to this view there is a bi-directional relationship between economic development and women's empowerment, defined as improving the ability of women to access the constituents of development, in particular health, education, earning opportunities, rights, and political participation (Duflo, 2016).

This study assesses the role of women's groups in Nigeria, with the hope of contributing to existing literature on the effectiveness and coordination of women's groups toward addressing the issue of low political participation by women. Osun state in South-West Nigeria is used as the case study. Presently, Osun state has the highest percentage of women in elected positions in Nigeria. The study made efforts to evaluate the role of women's groups in women's political participation in Osun state and examine problems that have made the women's groups (in) effective and (un) coordinated.

## The Women's Movement in Nigeria, the Journey so Far

The women's movement in Nigeria has come a long way, since the pre-colonial period to the present, in which Nigerian women have been organizing themselves to redefine their position in the society.

The umbrella body of all women-based organizations in Nigeria, the National Council of Women's Societies (NCWS), comprising a network of independent women's organizations was founded in 1959. The goals of the NCWS include, among others: the improvement of welfare; progress and the standard of living of women; and increasing the role of women in political life for more access to decision making (Olojede, 2000). Having been in existence for almost five decades, with membership spanning all regions of Nigeria, it has successfully given recognition to an unprecedented number of women in various spheres and from all walks of life.

Nigerian women have participated actively in all stages of the country's development beginning from the pre-colonial, through colonial to the post-colonial era. From records, women's struggles to correct acts of discrimination and violence dates back to the nineteenth century and they have actively participated in activities aimed to better their lot, in spite of the fact that such movements were not identified or labeled with any specific name at the earlier stage.

The significant role played by women in anti-colonial struggles, the struggles during the National Independence and social modernization, resulted in the formation of women's movements (Nigerian Group, 2011).

Women's interest was recognized in the pre-colonial era because of their importance in the distributive sector. Women were involved in trading activities in agriculture which was produced grown by men, and in craft and services. During this period women had a group consciousness and solidarity based on mutual interest and needs; they held important rights in the society, notably the right to discuss public policy, the right to representation on decision making bodies, and the right to property and inheritance. While women were not equal with men socially and politically they did, however, wield influence in policy making and processed institutional mechanisms for making the influence felt (Johnson, 1982). Colonialism, however, became a break which altered the existing position of women in African societies, including Nigeria. As observed by Ettienne and Lealock (1980) women's economic roles and their ability to participate in local government, in particular, were altered during this period. Within these changed circumstances of the colonial situation and the perceived threat to women's interests, women re-strategized by regrouping their forces to preserve and protect their interests. Strategies employed included organizing market women along new lines, using traditional skills and concepts of leadership, as well as western protest actions. Notable in this were the Southwestern Nigerian women, and in roughly the same period the Egba board of management in Abeokuta also came on board. This effort of women to protect their interest continued to mount even after the colonial period through regrouping in small clusters, mostly for their economic welfare. The four World Conferences on Women in 1975, 1980, 1985, and 1995, however, helped women in Nigeria to gain from the opportunities provided therein (Aluko, 2009). Notably, the experience of the Beijing Conference is worth mentioning here. Before the Beijing Conference in 1995, many women issues where left unaddressed, but soon afterwards a lot of women came into the limelight and it was time to start action for change. From this, a modest gain was achieved, for instance through the passage of the child right bill, violence against women, and so on. The participation of Nigeria in the Beijing Conference was an eye opener that gave birth to the National Commission for Women, which later translated into the Ministry of Women Affairs both at federal and State levels, the implication of which was that there are now 36 Commissioners in 36 states for the Ministry. Since the Beijing Conference in 1995 there has been gradual progress in the location of women in power decision making, which has also given rise to people's sensitivity to gender issues; duly, the National Gender Policy is reviewed every 5 years in Nigeria to assess the progress of the Beijing conference outcome. Women have also explored other UN accords for their benefits, especially the Convention on the Elimination of Discrimination against Women (CEDAW). The gender equality and women empowerment goal of the millennium development goal is not left out; efforts being made in the achievement of the millennium development Goal 3 has enlightened more men and enabled them to be part of the achievement including, more recently, Goal 5 regarding Sustainable development. In fact, the awareness about women's rights is now growing among men. All the above have been bases for demanding change in the lives of women in Nigeria.

There has been a successful setting of the women's agenda both in the public and private sectors due to the various efforts and activities of the women's movement. The evolution in the women's movement articulating women's rights is having implications for national policies, and advocacy work for women has made it possible for the decree on violence against women to be passed in six states in Nigeria. Also, building the capacity of women in the form of entrepreneurial training and the provision of micro credit facilities have empowered them to stand on their own, equipped with the capacity to contribute to the development of their various community, improving coalition among grassroots women. The economic empowerment of women has also resulted in social and political empowerment. The growing female participation in politics has increased the number of female Deputy Governors from one to three, and more women are now being given the opportunity to be part of decision making. Presently, statistics available from the Independent National Electoral Commission for the period 2015–2018 show that there are six deputy governors in six (6) states in Nigeria including: Osun (Mrs. Titilayo Laoye-Tomori); Ogun (Mrs.Yetunde Abosede Onanuga); Lagos (Dr Idiat Oluranti Adebule); Rivers (Ipalibo Gogo Banigo); Enugu (Mrs. Cecilia Ezeilo); and AkwaIbom (Mrs. Valerie Ebe).

### Constraints of the Women's Movement in Nigeria

Despite the enormous achievement of the women's movement in Nigeria, at the same time there exists constraints limiting its effectiveness. The lack of priority setting among activists in women's organizations, which has given rise to a segregated women's movement with different interests, is a major impetus for this study. Corruption in the country has also encouraged nepotism, mediocrity, and has led to conflict of interest within the movement. Although, even with little or no coordination, continuity, or sustainability, as well as no clear objectives, mission, or vision for some of the women's organizations, Madunagu (2008) observed that their existence have been characterized nevertheless as *ad hoc* bodies, and are useful when the need arises.

Financial support from international donors, which was a major source of funding for most women-based organizations in Nigeria has, in recent times since the 2008 global economic downturn, been suspended, posing a major threat to funding of their activities. These irregularities of getting grants, and sometimes the absence or shortage of funds, has been a major problem in carrying out programs contributing to the advancement of women's causes in Nigeria. The lack of education and enlightenment toward a significant political orientation among women and a lack of understanding concerning the interpretation of interventions have led to problems in getting constitutions regarding women adopted and putting women's issues on the agenda. It has not been easy getting people to accept the sermon of the women's movement; most times people lack interest and there is difficulty in getting women commitment to forming a strong union or alliance. Even those women's associations that have been formed are difficult in handling, as most times there is no confidence or trust in relating with each other in the movement. The lack of proper coordination among women's groups and associations has been a threat to the effectiveness of the women's movement in setting national gender policies in Nigeria. There are coalitions and networks among organizations in the movement but they do not function properly. Irregular meetings have been a major obstacle and this has also led to lack of documentation of NGOs activities within the movement.

## Methodology

**Figure 1:**
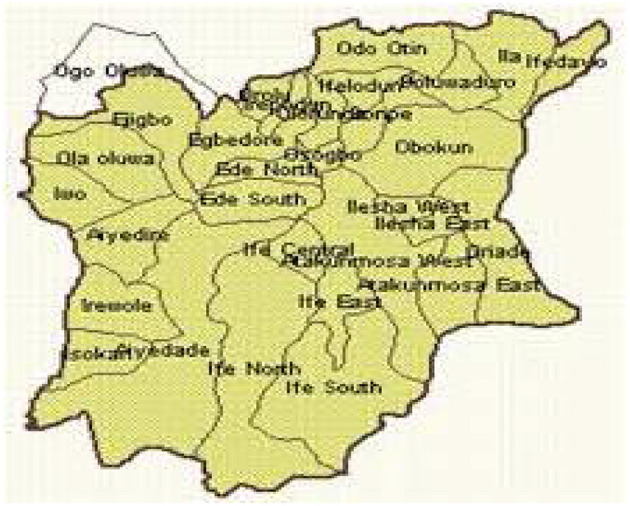
Map of Local Government Areas in Osun State, Nigeria.

The study was carried out using primary data from forums and dialogues with 6 women's groups consisting of an average of 30 members from 10 LGAs in Osun state, Nigeria. Both purposive and random sampling technique was employed, respectively, for the selection of the target population. The sample consisted of six women groups [National Council of Women Society (NCWS), Market Women Association, Widows Associations, Federation of Muslim Women Association of Nigeria (FOMWAN), Women Wing of Christian Association of Nigeria (WOWICAN) and the women's wing of the National Union of Teachers (NUT)] from ten (10) local governments, namely: Odo-Otin, Boripe/Boluwaduro, Ifedayo, Olorunda, Ede South, Ede North, Irewole, Ilesa West, Ife-Central, and Osogbo local governments. Permission was sought from all the women's group in the ten (10) LGAs to participate in their meetings. The women's groups were engaged in dialogue to elicit information on their awareness about existing National Gender Policy and Gender equality laws and policies, their perception about women's political participation, and their role in politics. Also, problems facing the women's groups were examined. Data collected were analyzed using descriptive method and content analysis. Also, explorative methods using existing literature were also employed (Figure 1).

### Ethical Considerations

Ethics approval was not required for this study as per applicable institutional guidelines and regulations. Oral informed consent was obtained from all participants. The leaders of the different women groups were informed of the study and their permission was sought and this was duly communicated to the members. Except for the name of the women groups; no direct or indirect identifiers of members were used. The rights and integrity of the respondents were respected in the course of the study. Participants were informed that participation in the study was voluntary and they were not compelled to provide information. Participants were free to withdraw from the study at any point they felt like. They were also made to understand that there were no risks associated with participating in the study.

## Discussion of Findings on the role of Women's group in Women Political Participation in Osun State

Having sought the permission of the leaders of the women groups to engage them in dialogue on the day of their meetings, it was duly communicated to the members. All the participants in the study participated voluntary. Attendance at the meetings comprised an average of 30 members each from the six (6) women groups in the ten (10) LGAs. Membership of all the women's groups cut across generations, ranging from young mothers in their early 20s to women in their 60s and over. The goals and objectives of most of the women's groups were mainly to cater for their members needs in their private sphere (household), especially their welfare, women empowerment, and poverty alleviation.

### Awareness of Existing National Gender Policy and Gender Equality Laws and Policies in Nigeria

From the investigation carried out, the evidence indicates that the awareness of the women groups on National Gender Policy, CEDAW, and other Gender Equality Laws are very low. Only a few of the women (5%) were aware of the 35% affirmative Action and most of the women, about 95%, were not aware of any existing gender policy, whether locally in Nigeria or International. Most of the women's groups lack political agenda, and their goals do not in any way align with any political agenda. Some of their goals include women empowerment, welfare promotion, poverty alleviation, career development, spiritual development, and so on (see Table 1).

**Table 1:** Table showing a Summary of Goals and Objectives of the Women Groups.

**S/N**	**Name of women group**	**Objectives/Goal**
1	National Council of Women Society (NCWS)	Advancement of women Issues
2	Market Women Association	Welfare and Poverty Alleviation
3	Widows Associations	Welfare and Poverty Alleviation Women Empowerment
4	Federation of Muslim Women Association of Nigeria (FOMWAN)	Spiritual development Welfare and Poverty Alleviation
5	Women Wing of Christian Association of Nigeria (WOWICAN)	Spiritual development Welfare and Poverty Alleviation
6	Women's wing of the National Union of Teachers (NUT)	Career development/Women Empowerment

*Source: Authors Compilation, 2018*.

### Perception on Women Political Participation and Their Role in Politics

Although most of the members were registered as voters, but not as constituents of any existing political party, only a very few are registered as party members. Also, members of women's groups accept political appointments, and also enjoy government's patronage when the opportunity arises.

A majority of the women revealed that they are not participating in politics due to a number of reasons which include among others, party meetings time, violence, unfavorable laws for Civil servants participation in politics, illiteracy, and a lack of support from men.

### Political Parties Meetings Time

According to the women, political party meetings are held mostly in the night and, to them, it is difficult for responsible women to attend such meetings. As a result, most of them shy away from being involved in politics. They, however, pointed out that a few women defy this norm; they are registered as party members and attend meetings but eventually they are stigmatized and labeled wayward and irresponsible by the society.

### Violence

The women's groups identified violence as a limiting factor for the women in participating in politics; they pointed to the manner in which politician use various violence means to scare women from participating in politics which the women described as fearful. As a result of the threats, most of the women lack the courage to go into politics.

### High Level of Illiteracy

A high level of illiteracy was a common trait among the majority of the women in the groups [Market Women Association, Widows Associations, Federation of Muslim Women Association of Nigeria (FOMWAN) and Women Wing of Christian Association of Nigeria (WOWICAN)], except for the women's wing of the NUT, where the lowest qualification amongst the women was a National Certificate of Education (NCE) and a National Council of Women Society (NCWS). Over 90% of the women in groups are not literate.

### Existing Laws and Civil Servants Participation in Politics

According to the women; existing laws are not favorable for civil servants in general, and women in particular, to participate in politics. Following the British tradition, the civil service in Nigeria continues to be characterized more or less by permanence, anonymity, and neutrality as contained in the 1999 Constitution (Iheme, 2003). As explained further by Iheme, permanence means that civil servants are career officers and ordinarily are expected to remain in the service for their entire working lives with different governments regimes. Thus, permanence ensures continuity. Anonymity means that civil servants are expected to work behind the scenes placing their skills and energies at the disposal of their political masters, who make the final decisions and receive applause for good work and jeers for bad work. While neutrality means that civil servants are prohibited from having political affiliations; they are expected to be faithful and impartially serve any government in power (Padfield, 1977; Adamolekun, 1986; Udoji, 1995; Iheme, 2003). This particular clause in Section 45 (1) (a) of the 1999 constitution has been a subject of controversy yet to be resolved.

### Other Constraints

The lack of support from men and the loss of values amongst female politicians, as well as a lack of funding for the groups, were also marked as common constraints challenging for all the women's groups.

The above constraints were major factors limiting the participation of all the women's groups in politics in the selected LGAs. The present low level of most women's groups' engagement in political matters in the LGAs Osun state makes it difficult for them to be involved in politics and contribute meaningfully to political issues at the local, state, and National levels. In 2019, which has already seen elections, a drastic measure is required.

## Conclusion

The study assessed the role of women's groups in women's political participation and economic development in Nigeria. Using Osun state as a case study, primary data was collected from forum and dialogues with 6 women's groups consisting of an average of 30 members from 10 LGAs. Findings indicate that the goals and objectives of the women's groups in the selected LGAs in Osun state are not politically inclined and the majority of their members only serve as voters during elections due to a number of constraints which include the women's lack of political right and awareness, late night political parties' meetings, violence, illiteracy, unfavorable laws on the participation of civil servants in politics, and so on. The knowledge within the women's groups on existing gender equality laws, both at the national and international levels, are limited. The study concludes that the present role played by women's groups at the grassroots level in Nigeria may not be adequate in encouraging more women's participation in politics, achieving its gender equality goals, and influencing economic development. It therefore becomes imperative to step up the activities of the women's groups to embrace political agenda and incorporate the same into their groups' goals and objectives if more women are to participate and be relevant in politics in elected positions in view of the 2019 elections. The study therefore suggests that more empowerment programs for women's groups, especially at the grassroots levels, by governments and all stakeholders should be a matter of priority and urgency in creating awareness among women's groups on the importance of participating in politics at the decision making level.

## Ethics Statement

Ethics approval was not required for this study as per applicable institutional and national guidelines and regulations. Oral informed consent was obtained from all participants.

## Author Contributions

The author confirms being the sole contributor of this work and has approved it for publication.

### Conflict of Interest Statement

The author declares that the research was conducted in the absence of any commercial or financial relationships that could be construed as a potential conflict of interest.
